# Neutral Fragment Filtering for Rapid Identification of New Diester-Diterpenoid Alkaloids in Roots of *Aconitum carmichaeli* by Ultra-High-Pressure Liquid Chromatography Coupled with Linear Ion Trap-Orbitrap Mass Spectrometry

**DOI:** 10.1371/journal.pone.0052352

**Published:** 2012-12-21

**Authors:** Jing Zhang, Zhi Hai Huang, Xiao Hui Qiu, Yi Ming Yang, Da Yuan Zhu, Wen Xu

**Affiliations:** 1 Lab of Chinese Materia Medica Preparation, The Second Clinical College of Guangzhou University of Chinese Medicine, Guangzhou, China; 2 Guangdong Provincial Academy of Chinese Medical Sciences, Guangzhou, China; University of New South Wales, Australia

## Abstract

A rapid and effective method was developed for separation and identification of diester-diterpenoid alkaloids (DDA) in the roots of *Aconitum carmichaeli* by ultra-high-pressure liquid chromatography coupled with high resolution LTQ-Orbitrap tandem mass spectrometry (UHPLC-LTQ-Orbitrap-MS^n^). According to accurate mass measurement and the characteristic neutral loss filtering strategy, a total of 42 diester-diterpenoid alkaloids (DDA) were rapidly detected and characterized or tentatively identified. Meanwhile, the proposed fragmentation pathways and the major diagnostic fragment ions of aconitine, mesaconitine and hypaconitine were investigated to trace DDA derivatives in crude plant extracts. 23 potential new compounds were successfully screened and characterized in *Aconitum carmichaeli,* including 16 short chain fatty acyls DDA, 4 N-dealkyl DDA and several isomers of aconitine, mesaconitine and hypaconitine.

## Introduction

*Aconitum carmichaeli* debx. (Fuzi), officially listed in the Chinese Pharmacopoeia, is one of the most widely used Chinese traditional medicinal herbs. It has been used for the treatment of rheumatism, neuralgia, and cardiac complaints for thousand years [1], [2]. However, the high toxicity risks and narrow therapeutic ranges limited the medicinal application on a larger scope. Typical symptoms of intoxication include rapid-onset facial and extremity paresthesias, chest discomfort, hypotension, and arrhythmias [3], [4]. Several cases of severe poisonings and fatalities due to ingestion of these diterpenoid alkaloids-containing prescriptions have been reported in many districts of the world [5], [6], [7]. For decades, the diterpenoid alkaloids originated from *A. carmichaeli* such as aconitine (AC), mesaconitine (MA) and hypaconitine (HA) have stimulated scientists’ strong interests in exploring their toxicity mechanisms and drug discovery [8], [9], [10]. QSAR analyses [11], [12], [13] and toxicology [14], researches indicated that these toxic reactions were mainly caused by the presence of the diester-diterpenoid alkaloids (DDA), the C_8_ and C_14_ diester-substituted diterpenoid alkaloids. Therefore, development of new reliable strategy for rapid detection and identification of new or minor bio-active diterpenoid alkaloids and toxins in *aconitum* plants is of great significance.

Previously, a number of analysis methods have been successfully applied to characterize diterpenoid alkaloids and their metabolites, including liquid chromatography (LC)-diode array detection (DAD) [16], LC-ESI-TOF/MS [17], LC-ESI-Ion Trap (IT)/MS [18], MALDI-TOF/MS [19] and FTICR (Fourier transform ion cyclotron resonance)-ESI-MS [20]. HPLC-TOF/MS shows unique advantages in providing metabolic profile acquisitions based on its low acquisition cycle time (from 0.1 to 0.3 s) and accurate mass measurement (average resolution of ∼5 ppm) [21]. Despite of more cycle time consumption, FTICR has been introduced as an alternative mass analysis tool to achieve better mass accuracy. Recently, Ultra-high-pressure liquid chromatography (UHPLC)-ESI-LTQ-Orbitrap high resolution tandem mass spectrometry analytical platform has spread thanks to the high chromatographic separation, ion scan sensitivity, and accurate mass measurement [22], [23]. UHPLC is a very powerful tool for fast and efficient separation of complex chemicals mixtures. The combination of Orbitrap technology with a linear ion trap has been shown to enable fast, sensitive and reliable detection and identification of small molecules regardless of relative ion abundance [24], [25]. This hybrid configuration provided greatly strengthened selectivity and suitability of different scan type according to different data demand. Furthermore, external calibration can be used to obtain high mass accuracy (<3 ppm) of all measured spectra resulting in a simplified experimental protocol.

In the present study, a simple and effective UHPLC-ESI-LTQ/Orbitrap MS^n^ method was developed to characterize the diester-diterpenoid alkaloids (DDA) in *A. carmichaeli*. The fragmentation pathways of different type DDA were studied by HR-MS^n^ and the diagnostic fragmentation behaviors were summarized. With the strategy of neutral fragment monitoring and diagnostic fragmentation filtering, a total of 42 DDA including 16 new type of short chain lipo-DDA were rapidly detected and identified by UHPLC-LTQ/Orbitrap-MS, as well as N-deethyl type DDA and other potential new structural components of DDA were reported for the first time.

## Materials and Methods

### Reagents and Chemicals

HPLC-grade acetonitrile, methanol and formic acid were purchased from Sigma Aldrich (St. Louis, MO, USA). Water (18.2 MΩ) was from a Milli-Q water system (Millipore, Bedford, MA, USA). The roots of *A. carmichaeli* were purchased from the GAP base in Jiangyou county, Sichuan province, China, and authenticated by Professor Zhihai Huang in our lab. Standards of aconitine (AC), mesaconitine (MA) and hypaconitine (HA) were obtained from National Institute for the Control of Pharmaceutical and Biological Products (NICPBP) (Beijing, China).

### Sample Preparation

Powdered root (2 g) was refluxing extracted in 30 mL of methanol at 80°C for 1 h and then diluted 10 times with methanol. The diluted solution was filtered through a 0.22 *µ*m membrane filter and directly analyzed by LC-MS.

### LC System

LC analyses were performed on a Thermo Accela UHPLC system (Thermo fisher Scientific, San Joes, CA, USA) consisting of a quaternary pump, a diode-array detector (DAD), an autosampler, and a column compartment. Samples were separated on a Waters AQUITY UPLC CSH C_18_ column (2.1×100 mm, 1.7 *µ*m) at room temperature. The mobile phase consisted of acetonitrile (A) and water containing 0.2% formic acid (B) and the elution gradient was set as follows: 15% A (0 min), 40% A (15 min) and 85% A (20 min). The mobile phase flow rate was 200 *µ*L/min and the injected volume was set at 1 *µ*L.

### Mass Spectrometry and Data Processing

For LC-ESI-MS^n^ experiments, a Thermo-fisher LTQ-Orbitrap XL hybrid mass spectrometer (Thermo Fisher Scientific, Bremen, Germany) was connected to the UHPLC instrument via an ESI interface. The conditions of ESI source were set as follows: Sheath and auxiliary gases flow, 25 and 3 arbitrary units, respectively; spray voltage, 4 KV; capillary temperature, 320°C and tube lens voltage: 120 V. Samples were analyzed in positive ion mode and the full scan mass range was set between *m/z* 350–1200 with the acquisition of centroided-type mass spectra. Accurate mass analyses were calibrated according to the manufacturer’s guidelines using a standard solution mix of sodium dodecyl sulphate, sodium taurocholate, the tetrapeptide MRFA acetate salt and Ultramark. In the full scan mode, resolution of the Orbitrap mass analyzer was set as 30,000 (FWHM as defined at *m/z* 400). In the MS^n^ experiments, data-dependent MS^n^ scanning was performed to minimize total analysis time as it can trigger fragmentation spectra of target ions and prevent repetition by dynamic exclusion settings. In the data-dependent scans, the FT resolution was 15, 000. The collision energy for collision-induced dissociation (CID) was adjusted to 40% of maximum, the isolation width of precursor ions was *m/z* 2.0 and default values were set for other CID parameters. A series of potential interference or contaminant ions were added to exclusion list to avoid triggering their MS^n^ data acquisitions. A syringe pump was used for the direct infusion of standard solutions (about 10 *µ*g/mL in methanol) in positive mode and flow rate was set at 10 *µ*L/min. Capillary temperature was set at 275°C, sheath gas 5 arbitrary units and no auxiliary gas was needed for direct infusion. All other parameters were identical to those in LC-MS^n^ experiments.

**Figure 1:**
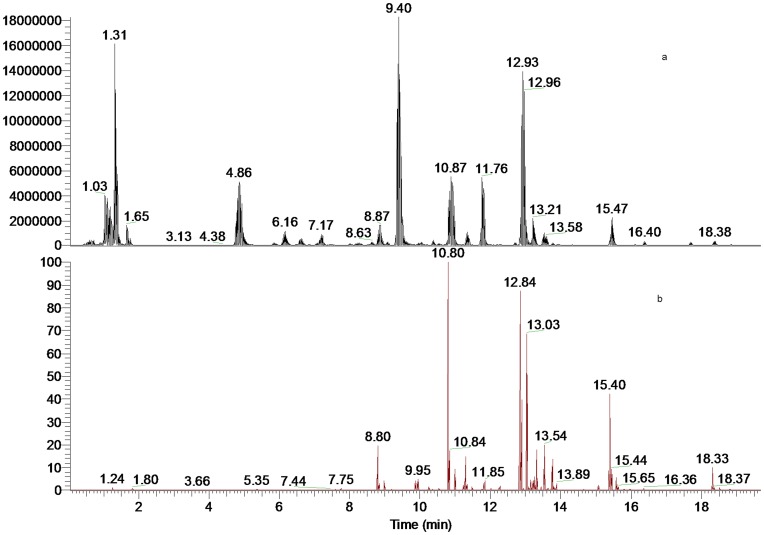
LC-MS spectra of the extract of daughter roots of *A. carmichaeli*. (a). Total ion chromatogram (TIC); (b). Neutral fragment chromatogram (NFC) for 60 Da.

The software of Mass Frontier 6.0 (Thermo Fisher Scientific) and Xcalibur 2.1 (Thermo Fisher Scientific) was employed for data analysis. After the automated removal of noise and baseline signals, the theoretical extract chromatogram of main DDA was shown in the plot of neutral fragment for *m/z* 60 (Figure 1b). Considering the possible elemental composition of potential components existed in *A. carmichaeli*, the number and types of expected atoms was set as follows: carbons≤100, hydrogens≤200, oxygens≤50, nitrogens≤5. The accuracy error threshold was fixed at 5 ppm.

## Results and Discussion

### MS^n^ Experiments

For LC-MS^n^ experiments, there are several options of fragmentation type provided by LTQ-Orbitrap analyzer (CID, HCD and PQD). With the benefit of linear ion trap, MS^5^ CID fragmentation data are easily acquired when ion abundance is adequate. In this study, three different fragmentation measurements were conducted in independent LC-MS^n^ experiments. In the first measurement the survey scan was performed in the Orbitrap analyzer (at R = 30,000) and was followed by MS^2^ scan in the Orbitrap (at R = 15,000). These high accurate tandem mass spectra can provide unequivocal evidences for identification of fragment and neutral loss. However, the high mass resolution and the scan rate were compromised in Orbitrap detection. In some cases, peaks were too narrow to produced sufficient data points for high resolution fragment data acquisition. To make up for it, other two separate measurements were performed, in which the CID MS^2^ and MS^3^ experiments were conducted by dynode detection, respectively. This faster acquisition of MS^n^ spectra could provide more fragment ions and the order of fragmentation could be determined to show evidence of structural identification.

The optimal CID collision energy was pre-tested by direct infusion experiments of reference compound hypaconitine (HA) and by LC-MS^n^ experiments of *A. carmichaeli* sample. In general, a low normalized collision energy of 30∼35% generated the minimal numbers of the fragments and the maximum intensity of fragment ions. In contrast, a 45% or higher energy made fragment ions abundances dropped significantly. As a result, compromised normalized collision energy of 40% was adopted in the LC-MS^n^ experiments.

### Fragmentation Pattern of Selective DDA Reference Compounds

**Figure 2:**
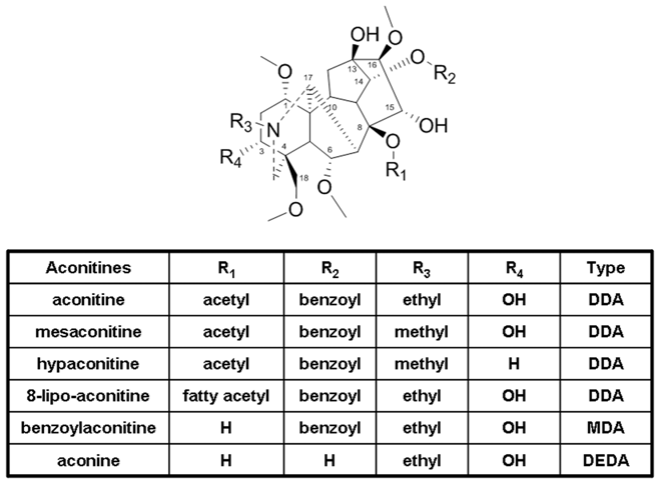
The structures of typical DDA, MDA and DEDA in *A. carmichael.*

The diterpenoid alkaloids in *A. carmichaeli* could be divided into three major groups, diester diterpenoid alkaloids (DDA), monoester diterpenoid alkaloids (MDA), and de-esterified diterpenoid alkaloids (DEDA) [26], according to the different substitution types at the site of C_8_ and C_14_ (Figure 2).

**Figure 3:**
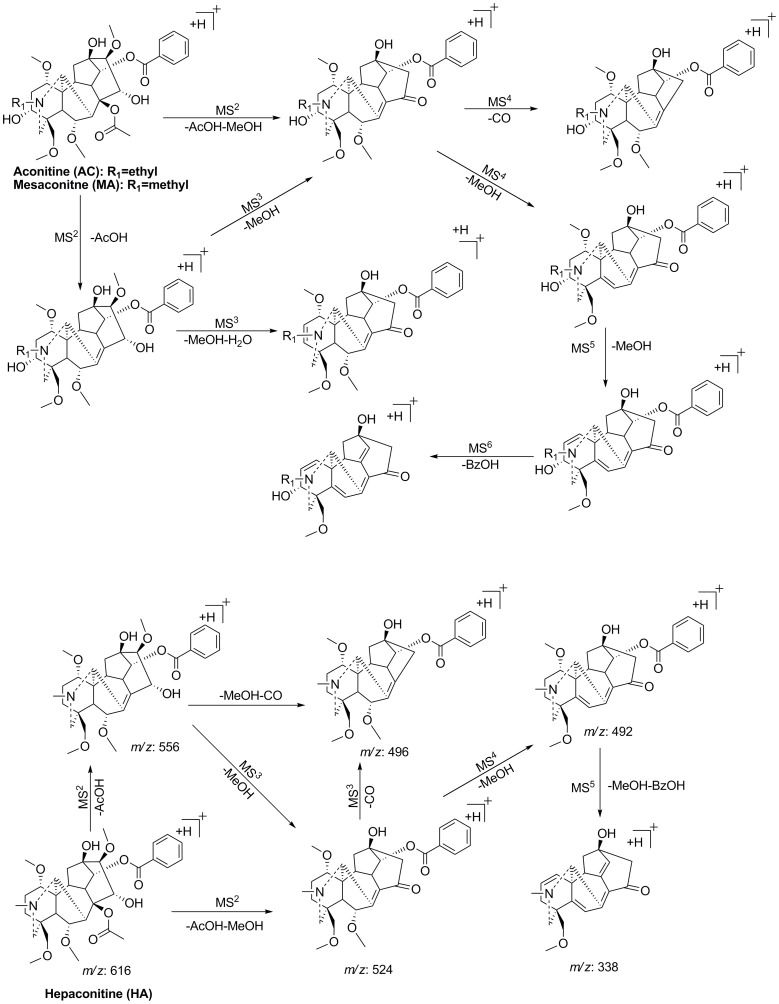
The key fragmentation patterns of aconitine, mesaconitine and hypaconitine.

The high-resolution tandem mass spectrometric analyses of typical DDA (mesaconitine (MA), hypaconitine (HA), and aconitine (AC)) were exhaustively investigated by direct infusion. Figure 3 summarized the common proposed fragmentation pathways of MA, HA and AC. According to the previously reported fragmentation pathways [19], [20], the neutral loss of 60 Da, 28 Da, 32 Da, 18 Da and 122 Da in MS^n^ spectra could be assigned as AcOH, CO, MeOH, H_2_O and BzOH, respectively. In their MS^2^ spectra the most abundant ion resulted from the elimination of a molecule of AcOH at C_8_ site, which could be a diagnostic neutral loss for the differentiation of DDA from monoester diterpenoid alkaloids (MDA) and de-esterified diterpenoid alkaloids (DEDA). In the MS^3^, MS^4^ and MS^5^ high resolution mass spectra, consecutive neutral losses of MeOH denoted the successive losses of three methoxy groups at C_1_, C_6_, C1_6_ or C_18_ sites from the skeleton. Chen *et al.* [27] used a quantum chemistry method to theoretically calculate the binding energies of the four methoxy groups and to predict the order of elimination of methanol between the four substituted sites. The order of the average bond energies of the four methoxy groups at C_1_, C_6_, C1_6_ and C_18_ sites is: C_16_< C_1_< C_6_< C_18_. Thus the first neutral loss of CH_4_O in the MS^2^ spectrum referred to the methoxy group at C_16_ site. The most prominent ion of [M+H-AcOH-MeOH-CO]^+^ in MS^n^ spectra, suggested that the most active elimination of MeOH occurred at C_16_ site and meanwhile could be a key fragmentation to determine the presence of 15-OH, where a neutral molecule of CO was eliminated when the enol form tautomerized into the ketone form [28]. For AC and MA, the ion of [M+H-AcOH-MeOH-H_2_O]^+^ in MS^2^ and MS^3^ spectra could be a diagnostic fragment to identify the hydroxyl group at C_3_ site, since the same fragment ion were not observed in MS^n^ spectra of HA. This fragmentation pattern was partly consistent with previous work [29], in which MS/MS fragmentations were conducted by Q-TOF analyzer.

### The Basic Strategy of Detection and Characterization of DDA in *A. carmichaeli*

**Figure 4:**
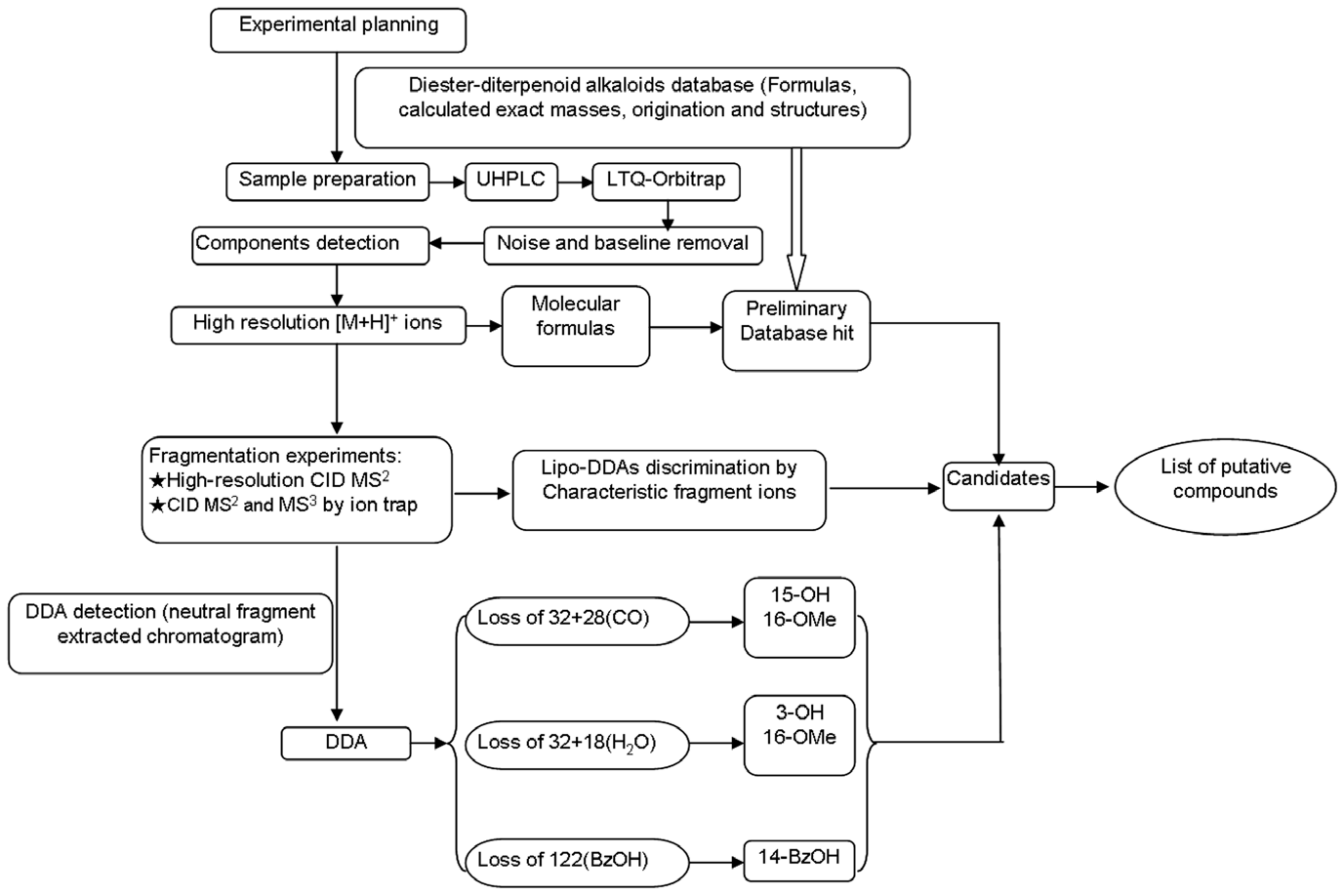
Schematic workflow of the automated data processing and rapid identification of DDA in *A. carmichaeli*.

A database was set to review all the DDA from *A. carmichaeli* and related species. Basically, major DDA found in *A. carmichaeli* show 15-OH, 3-OMe and 16-OMe substitutions, while those with 10-OH and 3-OH substituted were identified as well [30]. Based on the typical fragmentation behaviors described above, a strategy of rapid differentiation and identification of trace DDA was summarized in Figure 4. Firstly, main DDA constituents were preliminarily extracted in the high resolution full mass range scan, where the protonated ions of DDA were concentrated in the a mass range of 600–750 Da and the 8-acetyl DDA could be rapidly distinguished by neutral fragment extracted chromatogram (NFC) for 60 Da (Figure 1b). Then the substitution pattern of DDA could be determined by the diagnostic neutral losses of MeOH+CO and/or MeOH+H_2_O and their related neutral molecular. Lastly, the proposed fragmentation patterns were compared with reference compounds in the literatures [19], [20].

### UHPLC-ESI-MS^n^ Analysis of DDA from the Roots of* A. carmichaeli*

Figure 1 shows the UHPLC-MS total ion chromatogram (TIC) and neutral fragment chromatogram (NFC) of the roots of *A. carmichaeli*. As for the introduction of UHPLC system and small particle-size (1.7 *µ*m) column, the total analysis time for separation of *A.carmichaeli* was limited within 20 min with a high peak resolution, which was approximately four times faster than that of a conventional column packed with 5.0 *µ*m particles. By profiling the acetyl-DDA with neutral fragment extracted chromatogram (NFC) and characteristic fragment ions searching, at least 42 DDA were detected and characterized, many of which were potential new DDA (Table 1).

**Table 1:** Compounds identified in *A. carmichaeli.*

	*t*_R_	[M+H]^+^	Mass error (ppm)	Characteristic fragment ions	Identification
1	6.64	676.33411 (C_35_H_50_O_12_N)	1.36	MS^2^[676]: 572, 540, 512, 522, 480	8-hbt-benzoylmesaconine*
2	7.14	690.31573 (C_35_H_48_O_13_N)	3.71	MS^2^[690]: 556, 524, 496 MS^3^[556]: 524, 496, 464, 338	8-mlc-benzoylhepaconine*
3	7.46	602.29595 (C_32_H_44_O_10_N)	−0.02	MS^2^[602]: 542, 482, 510, 570, 324	N-demethyl-hypaconitine*
4	7.72	634.28870 (C_32_H_44_O_12_N)	2.90	MS^2^[634]: 574, 556, 524, 538	unknown
5	8.29	690.31604 (C_35_H_48_O_13_N)	4.02	MS^2^[690]: 572, 512, 522, 540 MS^3^[572]: 512, 540, 480, 522, 354	8-scn-benzoylmesaconine*
6	8.29	660.34186 (C_35_H_50_O_11_N)	4.02	MS^2^[660]: 556, 524, 496 MS^3^[556]: 524, 496, 464, 338	8-hbt-benzoylhepaconine*
7	8.57	688.29877 (C_35_H_46_O_13_N)	2.40	MS^2^[688]: 572,512, 540 MS^3^[572]: 512, 540, 480, 522, 354	8-fmr-benzoylmesaconine*
8	8.64	690.35168 (C_36_H_52_O_12_N)	3.28	MS^2^[690]: 586, 526, 554 MS^3^[586]: 526, 554, 536, 494, 368	8-hbt-benzoylaconine*
9	8.80	648.30505 (C_33_H_46_O_12_N)	3.59	MS^2^[648]: 588, 556, 538, 598, 528 MS^3^[588]: 528, 538, 524, 556m	10-OH-mesaconitine
10	9.26	630.29229 (C_33_H_44_O_11_N)	1.40	MS^2^[630]: 570, 598, 538, 612	unknown
11	9.84	632.30690 (C_33_H_46_O_11_N)	0.36	MS^2^[632]: 572, 512, 540 MS^3^[572]: 512, 540, 494, 480	10-OH-hepaconitne/iso
12	9.96	618.29140 (C_32_H_44_O_11_N)	0.51	MS^2^[618]: 558, 526, 536, 494, 508 MS^3^[558]: 498, 526, 462	N-deethyl-aconitine*
13	10.04	660.34271 (C_35_H_50_O_11_N)	4.87	MS^2^[660]: 556, 524, 496 MS^3^[556]: 524, 496, 464, 338	8-hbt-benzoylhepaconine*
14	10.08	602.29704 (C_32_H_44_O_10_N)	1.07	MS^2^[602]: 542, 510, 482, 570	N-demethyl-10-OH-13-deoxy-hypaconitine*
15	10.16	704.33063 (C_36_H_50_O_13_N)	2.96	MS^2^[704]: 586, 526, 554 MS^3^[586]: 526, 554, 536, 494, 368	8-scn-benzoylaconitne *
16	10.20	632.30707 (C_33_H_46_O_11_N)	0.53	MS^2^[632]: 572, 540, 600, 512, 354	10-OH-hepaconitne/iso
17	10.20	674.32019 (C_35_H_48_O_12_N)	3.09	MS^2^[674]: 556, 524, 496 MS^3^[556]: 524, 496, 464, 338	8-scn-benzoylhepaconine*
18	10.42	672.30481 (C_35_H_46_O_12_N)	3.36	MS^2^[672]: 556, 524, 496 MS^3^[556]: 524, 496, 464, 338	8-fmr-benzoylhepaconine*
19	10.50	616.31274 (C_33_H_46_O_10_N)	1.12	MS^2^[616]: 556, 524, 496, 492	10-OH-13-deoxyhepaconitine*
20	10.50	702.35242 (C_37_H_52_O_12_N)	4.01	MS^2^[702]: 556, 524, 496 MS^3^[556]: 524, 496, 464, 338	8-adp-benzoylhepaconine*
21	10.82	632.30994 (C_33_H_46_O_11_N)	3.40	MS^2^[632]: 572, 540, 512, 522 MS^2^[572]: 512, 540, 522, 480, 354	mesaconitine
22	11.13	702.31567 (C_36_H_48_O_13_N)	3.65	MS^2^[702]: 586, 526, 554 MS^3^[586]: 526, 554, 536, 494, 368	8-fmr-benzoylaconine*
23	11.20	646.32255 (C_34_H_48_O_11_N)	0.36	MS^2^[646]: 586, 554, 536 MS^3^[586]: 536, 554, 522	10-OH-15-deoxyaconitine*
24	11.30	662.32117 (C_34_H_48_O_12_N)	4.07	MS^2^[662]: 602, 570, 612, 542, 552 MS^3^[602]: 542, 570, 552, 384	aconifine
25	11.50	646.32273 (C_34_H_48_O_11_N)	0.54	MS^2^[646]: 554, 586, 522, 490	unknown
26	11.52	702.31610 (C_36_H_48_O_13_N)	4.08	MS^2^[688]: 572,512, 540 MS^3^[572]: 512, 540, 480, 522, 354	8-gtn-benzoylmesaconine*
27	11.81	616.31360 (C_33_H_46_O_10_N)	1.98	MS^2^[616]: 556, 524, 506 MS^3^[556]: 506, 524, 492	15-deoxymesaconitine
28	11.85	614.29529 (C_33_H_44_O_10_N)	−0.68	MS^2^[614]: 554, 494 MS^3^[554]: 494, 372, 522	2,3-didehydrohypaconitine*
29	12.18	688.33667 (C_36_H_50_O_12_N)	3.92	MS^2^[688]: 570, 538, 510MS^3^[570]: 510, 538, 478, 352	8-scn-benzoyl-3-deoxyaconine*
30	12.28	602.29711 (C_32_H_44_O_10_N)	1.14	MS^2^[602]: 542, 510, 478, 570, 492	N-demethyl-15-deoxy-mesaconitine*
31	12.90	616.31197 (C_33_H_46_O_10_N)	0.35	MS^2^[616]: 556, 524, 584, 496MS^3^[556]: 524, 496, 338	Hypaconitine
32	13.18	646.32272 (C_34_H_48_O_11_N)	0.53	MS^2^[646]: 586, 526, 554, 536 MS^3^[686]: 526, 554, 536, 494, 368	Aconitine
33	13.25	630.32861 (C_34_H_48_O_10_N)	1.34	MS^2^[630]: 570, 538, 520	Indaconitine
34	13.54	600.31685 (C_33_H_46_O_9_N)	0.14	MS^2^[600]: 540, 508, 480 MS^3^[540]: 480, 508, 458	Isodelphinine
35	13.75	628.31207 (C_34_H_46_O_10_N)	0.45	MS^2^[628]: 568, 508, 536 MS^3^[568]: 508, 536, 386	3-dehydroaconitine*
36	13.88	600.31682 (C_33_H_46_O_9_N)	0.12	MS^2^[600]: 540, 508, 568 MS^3^[540]: 508	delphinine
37	15.08	614.33211 (C_34_H_48_O_9_N)	−0.24	MS^2^[614]: 554, 522, 536	Chasmaconitine
38	15.42	630.32733 (C_34_H_48_O_10_N)	0.06	MS^2^[630]: 570, 538, 510	3-deoxyaconitine
39	15.61	600.31964 (C_33_H_46_O_9_N)	2.93	MS^2^[600]: 540, 522, 480, 482 MS^3^[540]: 480, 522, 462, 508	unknown
40	15.84	672.34119 (C_36_H_50_O_11_N)	3.35	MS^2^[672]: 554, 522, 494	8-scn-benzoyl-3,13-deoxyaconitine*
41	17.34	660.34174 (C_35_H_50_O_11_N)	3.90	MS^2^[660]: 572, 512, 522, 540 MS^3^[572]: 540, 512, 522, 480, 354	8-btn-benzoylmesaconine*
42	18.35	614.33265 (C_34_H_48_O_9_N)	0.29	MS^2^[614]: 554, 522 MS^3^[554]: 494, 522, 458	3, 13-deoxyaconitine

Mlc, hbt, scn, fmr, adp, gtn and btn represent the residues of malic acid, hydroxybutanoic acid, succinic acid, fumaric acid, adipic acid, glutaconic acid and butanoic acid, respectively.

* Denotes the potential new alkaloids.

### Isomers of HA, MA and AC and their Derivatives

**Figure 5:**
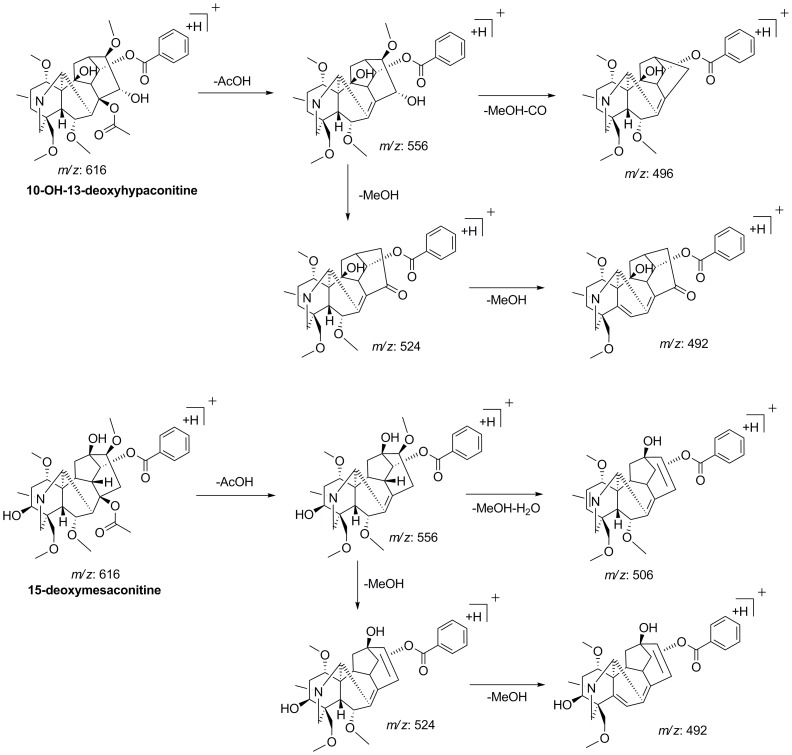
The key fragmentation pathway of two isomers of hypaconitine.

Peaks of 31, 21 and 32 were unambiguously identified as hypaconitine (HA), mesaconitine (MA) and aconitine (AC) respectively, which were confirmed by comparing their *t*_R_ values and mass spectra with those of standards. Two isomers of HA (peak 19 and 27) were detected at *t*_R_ 10.50 and 11.81 min, both of which showed the [M+H]^+^ ions at *m/z* 616.3127 with the elemental composition of C_33_H_46_O_10_N. The base ion at *m/z* 556.2891 (C_31_H_42_O_8_N, −2.47 ppm) in MS^2^ spectrum indicated that they were DDA derivatives. For peak 19, the characteristic [M+H-AcOH-MeOH-CO]^+^ ion at *m/z* 496.2682 (C_29_H_38_O_6_N, −2.34 ppm) observed in MS^2^ proved the presence of C_15_-OH substitution, while the [M+H-AcOH-MeOH-H2O]^+^ ion at *m/z* 506 wasn’t detected, suggesting that there was no hydroxyl group occupied at C_3_ site. The key fragmentation behaviors were contrary for peak 27. The presence of a hydroxyl group at C_3_ were resulted from the loss of [M+H-AcOH-MeOH-H_2_O]^+^ in MS^2^ and MS^3^ spectra to generate fragment ion at *m/z* 506.2530 (C_30_H_36_O_6_N, −1.41 ppm), while the possibility of C_15_-OH substitution was excluded due to the absence of the neutral loss of MeOH+CO (60 Da) at *m/z* 496 in MS^n^. By reviewing the literatures [31], the hydroxyl group substitution was most likely occurred at C_10_ position. Thus, peak 19 and 27 were identified as 10-OH-13-deoxyhypaconitine and 15-deoxymesaconitine, respectively and their proposed fragmentation pattern were illustrated at Figure 5.

**Figure 6:**
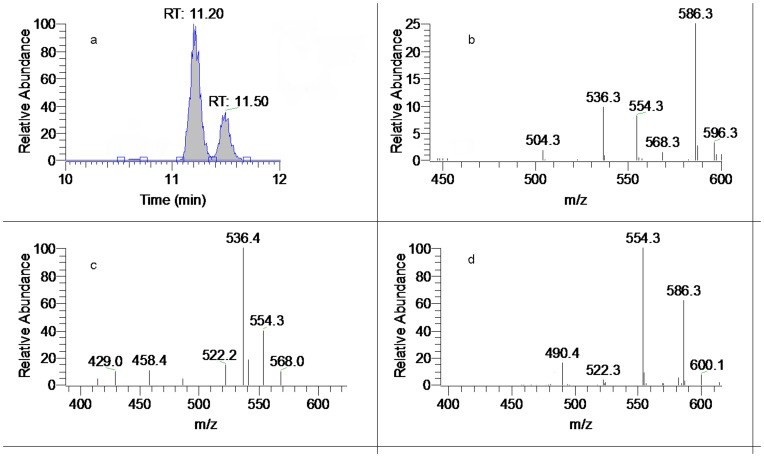
Mass spectra of peak 23 and 25. ( a). Extracted ion chromatogram (EIC) of *m/z* 646 (amplified); (b). MS^2^ of peak 23 (*t*_R_: 11.20 min); (c). MS^3^ of peak 23; (d). MS^2^ of peak 25.

So far, no isomer of AC has been found in *A. carmichaeli*. By means of diagnostic neutral loss screening and high-definition mass measurement, two isomers of aconitine were detected in *A. carmichaeli* in this study. Both peak 23 (*t*_R_: 11.20 min) and 25 (*t*_R_: 11.50 min) showed the identical [M+H]^+^ precursor ion at *m/z* 646.3227 (C_34_H_48_O_11_N). Comparing with AC, peak 23 exhibited very similar MS^n^ spectra, except that the key neutral loss of [M+H-AcOH-MeOH-CO]^+^ for identification of C_15_-OH was not observed. The MS^2^ spectrum showed a base ion at *m/z* 586.2994 (C_32_H_44_O_9_N, −2.86 ppm), as well as the [M+H-AcOH-MeOH]^+^ ion at *m/z* 554.2739 (C_31_H_40_O_8_N, −1.65 ppm) and [M+H-AcOH-MeOH-H_2_O]^+^ ion at m/z 536.2623 (C_31_H_38_O_7_N, −3.74 ppm). We deduced that peak 23 was a 15-deoxy DDA and the hydroxyl group was substituted to C_10_ position. Thus peak 23 was tentatively identified as 10-OH-15-deoxyaconitine, which was a potential new aconitine-type C_20_-diterpenoid alkaloid [26]. In the MS^2^ spectra of most DDA, the most abundant ions were [M+H-AcOH]^+^ fragments resulting from the elimination of a molecule of AcOH at C_8_ site. However the MS^2^ spectrum of peak 25 was so peculiar that the base ion was [M+H-AcOH-MeOH]^+^ at m/z 554.2737 (C_31_H_40_O_8_N, −1.99 ppm), although the crucial fragment ion of [M+H-AcOH]^+^ at *m/z* 586.2994 (C_32_H_44_O_9_N, −2.86 ppm) was also found as an prominent ion in MS^2^ (Figure 6). In addition, consecutive losses of MeOH (32 Da) were observed only, without any trace of the dehydration fragment to show hydroxyl group substitution in C_3_ or C_15_ position. Due to the lack of reference compounds and data from previous literature, its structure was not elucidated.

**Figure 7:**
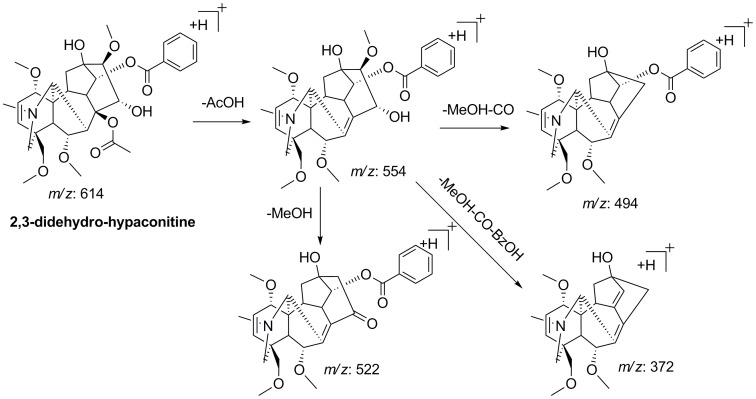
Proposed fragmentation pattern of 2,3-didehydro-hypaconitine.

Peak 11 and 16 both displayed a [M+H]^+^ ion at *m/z* 632.3070 (C_33_H_46_O_11_N), suggesting they were MA isomers. Their MS^2^ spectra all gave *m/z* at 572 as base peak and the loss of 120 Da may consist of an AcOH, MeOH and CO groups as mentioned above. Therefore, peak 11 and 16 were characterized as 10-OH-hypaconitine isomers.

In the extracted ion chromatogram (EIC) of *m/z* 614, there were three peaks (*t*_R_ 11.85 (peak 28), 15.08 (peak 37) and 18.35 min (peak 42)). In the FT full scan spectra, peak 37 and 42 shared the identical elemental composition of C_34_H_48_O_9_N and further yielded the same fragment ion at *m/z* 554, suggesting these to be DDA derivatives. The key fragment ion of [M+H-AcOH-MeOH-CO]^+^ were detected only in the MS^n^ spectrum of peak 42, which represented that their different substitution patterns at C_15_ site. There was no any sign of neutral elimination of MeOH+H_2_O for both peak 37 and 42. By investigating the reference data, peak 37 and 42 were identified as chasmaconitine and 3, 13-deoxyaconitine, the fragmentation pathways of which were reported previously [20].

Peak 28 showed the [M+H]^+^ precursor ion at *m/z* 614.2953 (C_33_H_44_O_10_N) and gave value of the ring and double-bond equivalent (RDB) of 12.5, which was 1 more than that of all other DDA (RDB  = 11.5) detected in *A. carmichaeli*. It was preliminarily assumed that peak 28 was the dehydration product of MA (C_33_H_46_O_11_N). Its MS^n^ spectra displayed the typical fragmentation behaviors of DDA, in which the neutral losses of acetic acid, MeOH and CO were observed, as well as the loss of BzOH. Neither the [M+H-AcOH-H_2_O]^+^ nor [M+H-AcOH-MeOH-H_2_O]^+^ product ion was detected in the MS^2^ spectrum, which allowed us to infer that the elimination of H_2_O could be assigned to C_3_ position to form 2,3-didehydro-hypaconitine [32]. With no further information, peak 28 was tentatively characterized as 2,3-didehydro-hypaconitine and its proposed fragment pathway was shown in Figure 7.

**Figure 8:**
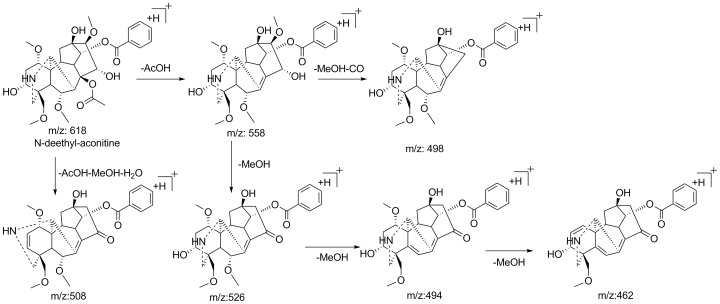
Proposed fragmentation pattern of N-deethyl-aconitine.

A pair of deoxyaconitine isomers (peak 33 and 38) was detected in the EIC of *m/z* 630. They both exhibited [M+H]^+^ ion at *m/z* 630.3273 (C_34_H_48_O_10_N) and their MS^2^ spectra all gave ion at *m/z* 570.3051 (C_32_H_44_O_8_N, −1.83 ppm) as the base peak. On the basis of the characteristic fragment ion at *m/z* 510.2834 [M+H-AcOH-MeOH-CO]^+^, peak 33 and 38 could be easily discriminated as 13-deoxyaconitine (indaconitine) and 3-deoxyaconitine, respectively. Besides, a pair of isomers eluted at 13.54 and 13.88 min and they were tentatively identified as isodelphinine or delphinine [29].

The peaks at 8.80 min (peak 9) and 11.30 min (peak 24) had the similar fragmentation pathways with MA and AC, respectively. According to the MS^2^ data and the literature reports [28], it could be deduced that peak 9 and 24 corresponded to 10-OH-mesaconitine and 10-OH-aconitine (aconifine), respectively. Other alkaloids such as peak 4, 10 and 39 were also characterized as new DDA, however their exact structures were not confirmed due to lack of literature support.

**Figure 9:**
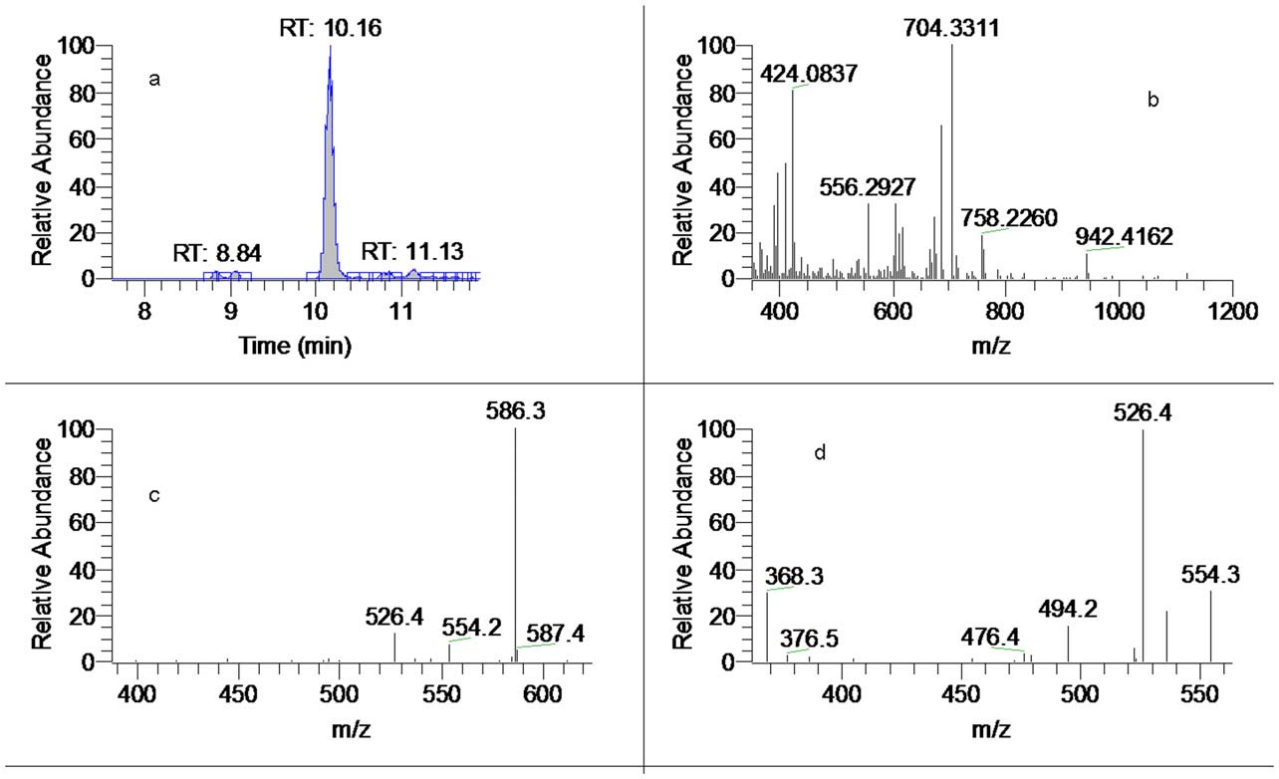
The mass spectra of peak 15 (8-scn-benzoylaconine). ( a). Extracted ion chromatogram (EIC) of *m/z* 704; (b). Full scan mass spectrum of peak 15; (c). MS^2^ spectrum of peak 15; (d). MS^3^ spectrum of peak 15.

### N-dealkyl-type DDA

Several N-dealkyl aconitine-type alkaloids such as N-deethyltalatisamine [33], N-deethylchasmanine [34] and 10-dehydroxyflavaconitine [35] were identified from *A. carmichaeli* and other species of *Aconitum*. However, no N-dealkyl-type DDA has been reported in *A. carmichaeli* so far. In this study, new N-dealkyl aconitine-type of DDA were plausibly characterized by means of high resolution mass spectrometry and key neutral loss patterns. For instance, peak 12 exhibited [M+H]^+^ ion at *m/z* 618.2914 (C_32_H_44_O_11_N, 0.51 ppm), which showed less elemental composition of C_2_H_4_ than that of AC. Its MS^2^ spectrum gave a base fragment ion at *m/z* 558.2685 (C_30_H_40_O_9_N, −1.26 ppm, [M+H-AcOH]^+^), consistent with it being a diester aconitine-type diterpenoid alkaloid. The successive losses of 32 to produce ions at *m/z* 526.2427, 494.2150 and 462.1888 suggested the methoxy groups linked to C1, C6 and C16 sites. In the MS^3^ spectrum, two expected characteristic ions at *m/z* 508 and 498, derived from neutral loss of 50 Da (MeOH+H_2_O) and 60 Da (MeOH+CO) respectively, were observed. Based on its fragmentation pattern (Figure 8), Peak 12 was preliminarily identified as N-deethyl-aconitine, the de-esterification product of which has been reported from *A. carmichaeli* [36].

**Figure 10:**
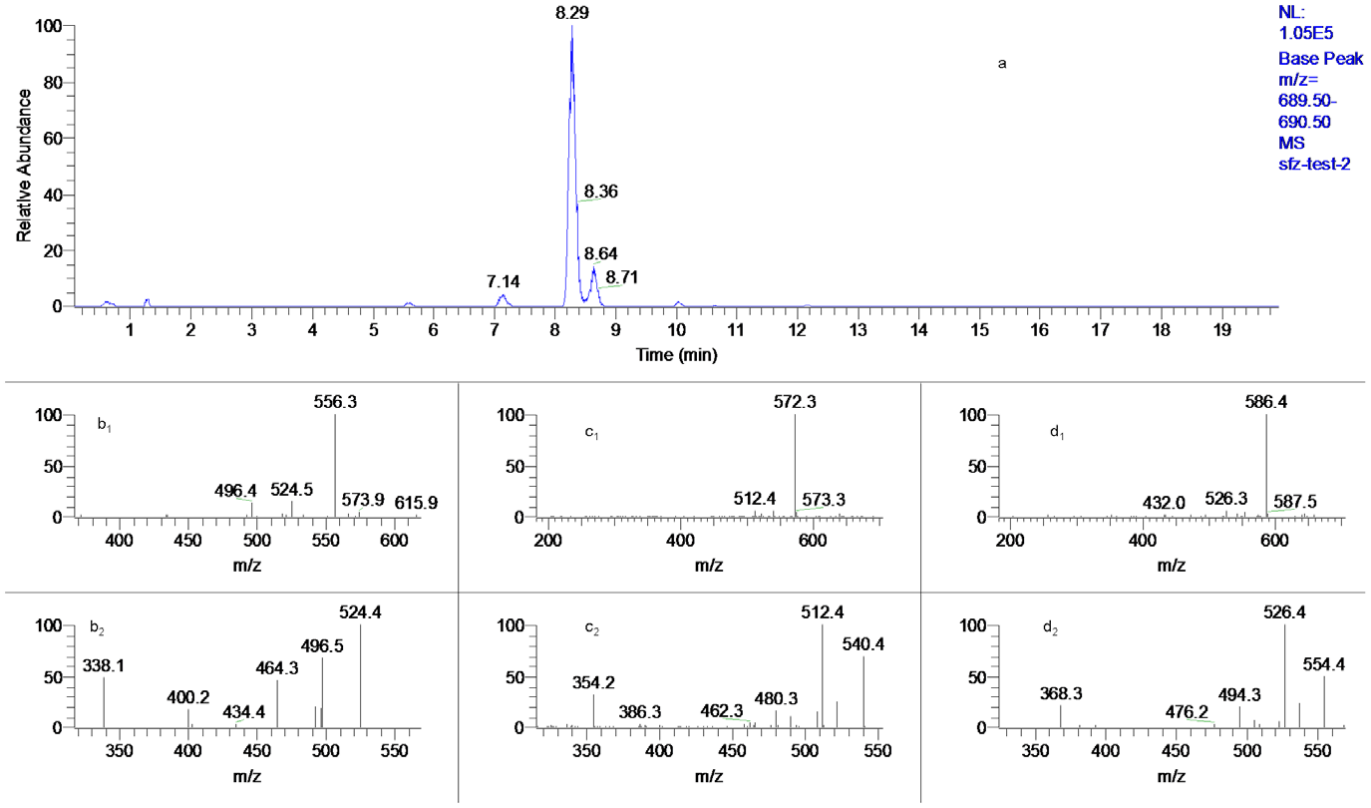
The mass spectra of peak 2, 5 and 8. (a). EIC of *m/z* 690; (b_1_). MS^2^ of peak 2; (b_2_). MS^3^ of peak 2; (c_1_). MS^2^ of peak 5; (c_2_). MS^3^ of peak 5; (d_1_). MS^2^ of peak 8; (d_2_). MS^3^ of peak 8.

In addition, three peaks (peak 3, 14 and 30) all gave a [M+H]^+^ ion at *m/z* 602.2970 (C_32_H_44_O_10_N). These three isomers could be deduced as the isomers of N-demethyl-hypaconitine by their MS^n^ spectra. Based on the key neutral loss, they were tentatively characterized as N-demethyl-hypaconitine, N-demethyl-10-OH-13-deoxyhypaconitine and N-demethyl-15-deoxy-mesaconitine, respectively (Table 1).

### Short Chain Fatty Acid Esters of DDA

Lipo-alkaloids, in which the C_8_ position is occupied by a long chain fatty acyl (14–25 carbon atoms) have been isolated and identified from *A. carmichaeli* [20], [37]. In this work, DDA with short chain fatty acyls (4–6 cabon atoms) or diacids residues were reported for the first time. Basically, the MS^n^ spectra of the lipo-DDA were identical with that of acetylated counterparts at C_8_ position and the base ions in the MS^2^ spectra were corresponded to the neutral losses of fatty acids. Typical base ions in the MS^2^ were at *m/z* 586, 572, 556, 570 and 554, corresponding to the skeletons of benzoylaconine, benzoylmesaconine, benzoylhypaconine, benzoyldeoxyaconine and benzoyl-3,13-deoxyaconine, respectively [20]. Therefore, the skeletons could be characterized by comparing their MS^n^ spectra with those of acetylated DDA and the type of fatty acid could be determined by acquiring high-resolution full scan mass spectra. For example, CID of *m/z* 704 at *t*_R_ 10.16 min produced identical MS^2^ and MS^3^ spectra with that of AC to give characteristic fragment ions at *m/z* 586, 526, 536, 494 and 368 (Figure 9), indicating that it was derived from aconine. The neutral loss of C_4_H_6_O_4_ were determined by high resolution mass of [M+H]^+^ at *m/z* 704.3311 (C_36_H_50_O_13_N), corresponding to the elimination of succinic acid. Therefore, the ion at *m/z* 704 corresponded to 8-scn-benzoylaconine (here scn denotes succinic acid).

Three peaks gave the same protonated ion at *m/z* 690 (*t*_R_: 7.14, 8.29 and 8.64 min) (Figure 10) and their MS^2^ spectra exhibited different prominent fragment ions at *m/z* 556, 572, and 586, respectively, suggesting their were originated from HA, MA and AC, respectively. These arrangements were also confirmed by comparing their MS^3^ spectra with those of HA, MA and AC, respectively. The losses of malic acid (mlc), succinic acid (scn) and hydroxybutanoic acid (hbt) were confirmed by high resolution mass spectrometry. Thus peak 2, 5 and 8 were tentatively characterized as 8-mlc-benzoylhypaconine, 8-scn-benzoylmesaconine and 8-hbt-benzoylaconine, respectively. By using this method, the new finding of other twelve short chain lipo-DDA (peak 1, 6, 7, 13, 17, 18, 20, 22, 26, 29, 40 and 41) in which C_8_ was occupied by a 4C, 5C or 6C fatty acid, hydroxyl fatty acid or diacid were demonstrated and they have not been reported previously in *Aconitum* genus.

### Conclusion

In this study, a simple and rapid UHPLC-LTQ-Orbitrap-MS^n^ method has been successfully developed for separation and identification of diester-diterpenoid alkaloids in the roots of *Aconitum carmichaeli*. Based on the provided strategy, forty-two diester-diterpenoid alkaloids were extracted and characterized. A number of new diester-diterpenoid alkaloids including sixteen short chain fatty acid esters of DDA and four N-dealkyl-type DDA were detected. The UHPLC-LTQ-Orbitrap-MS^n^ method presented here provided essential data for further pharmacological and toxicological studies of *Aconitum* plants. Moreover, LC-LTQ-Orbitrap mass spectrometry has been demonstrated to be an effective tool for the analysis of the components and searching for some novel compounds in a complex plant extract.
